# Pubertal timing in adolescents with ADHD: extension and replication in an all-female sample

**DOI:** 10.1007/s00787-023-02239-z

**Published:** 2023-05-28

**Authors:** Emily A. Rosenthal, Stephen P. Hinshaw

**Affiliations:** 1 Department of Psychology, University of California, Berkeley, 2121 Berkeley Way, Berkeley, CA 94720-1650 USA; 2 Department of Psychiatry and Behavioral Sciences, UCSF Weill Institute for Neurosciences, University of California, San Francisco, 675 18th Street, San Francisco, CA 94107 USA

**Keywords:** ADHD, Puberty, Stimulants, Menarche, Pubertal timing

## Abstract

Pubertal timing predicts a miscellany of negative mental and physical health outcomes. Prior work examining pubertal timing in youth with attention-deficit hyperactivity disorder (ADHD) has failed to investigate potential sex specificity of results. Therefore, we aim to extend past findings in a sample of female adolescents with ADHD. We compare pubertal timing (1) between females with and without carefully diagnosed ADHD and (2) between females with ADHD who do vs. do not have a history of stimulant medication use during childhood. We examine 127 adolescent females with childhood-diagnosed ADHD and 82 matched neurotypical peers (*M*_age_: 14.2 years, *range:* 11.3–18.2) from the Berkeley Girls with ADHD Longitudinal Study (Wave 2). We measured pubertal timing using self-reported Tanner staging and age at menarche. Three strategies compared pubertal timing across groups: (1) ${\chi }^{2}$ tests of Tanner Stages, (2) *t* tests of residuals of pubertal status regressed on age, and (3) *t* tests of age at menarche. Pubertal timing of girls with and without ADHD did not differ significantly across methods and measures. Yet females with ADHD who had received stimulant medication during childhood menstruated later than those without a stimulant history, potentially related to differences in BMI across groups. On the other hand, no significant differences between medicated vs. non-medicated participants emerged for the two Tanner staging indicators. Our findings extend prior work, suggesting that females with ADHD are developing physically at a similar time as their peers, which parallels findings from previous mixed-sex samples that did not examine effects separately by sex.

## Introduction

Adolescence occurs during the second decade of life and marks the period of physical, cognitive, and social maturation between childhood and adulthood [1]. Across adolescence, youth seek and are given increasing autonomy and independence in selecting and shaping their environments; more time is spent with peers relative to parents [1, 2]. Risk taking and sensation seeking often increase, as do emotional responses to social contexts and the subjective experience of emotional intensity [2, 3]. In parallel, increases in abstract thinking about oneself and one’s long-term goals contribute to the development of identity [1]. Given the profound emotional, neurobiological, hormonal, cognitive, physical, and social changes that occur during adolescence, this period of the life span has been highlighted as a critical period for both risk and resilience [3].

One of the major drivers of change during adolescence is puberty. The pubertal transition involves three endocrine events: activation of the adrenal axis (adrenarche), activation of the gonadal axis (gonadarche), and activation of the growth axis, all of which are linked to considerable hormonal changes [4, 5]. (1) Adrenarche occurs at age 6–9 years in females and 7–10 years in males, leading to an increase in the concentration of adrenal steroid hormones. The result is the eventual growth of pubic hair, development of acne, and skeletal maturation [5, 6]. (2) Gonadarche begins approximately 1–2 years after adrenarche, at a mean age of 11 in females (range: 8–14) and 12 in males (range: 9–15); this hormonal event is believed to indicate the onset of puberty [5, 6]. It involves activation of gonadotropin-releasing hormone, which leads to increases in the concentration of testosterone in males and estrogen in females. This lengthy process (4–5 years) produces the gradual development of testes, facial hair, and voice changes in males, and of breast growth and eventual menstruation in females [5, 6]. (3) During the latter half of the pubertal process, activation of the growth axis is linked to a rise in growth hormone (plus sex steroids), contributing to a pubertal growth spurt, which occurs about 1–2 years later in males than in females [5, 6]. Overall, these hormonal and bodily changes contribute to patterns of cognition, emotion regulation, socialization, and risk taking that characterize adolescence [4, 7].

Pubertal development is often measured using self- or parent-reported physical development. One common metric is Tanner Line Drawings, which ask parents, clinicians, or youth to identify the youth’s physical development on one of a series of five progressively more developed drawings ranging from prepubertal to fully developed [8, 9]. For females, there is one drawing for breast growth and one for pubic hair growth. For males, there are separate drawings for pubic hair and testicular development. Prior work has established a moderately strong but imperfect correspondence among parent, clinician, and self-reported physical development [10, 11].

Although age is clearly correlated with pubertal development, the two are not synonymous. In fact, there is often a wide range in the ages at which youth reach physical milestones relative to same-sex peers (pubertal timing). It is important to note that pubertal timing shifts dynamically across the pubertal process depending on both the rate (tempo) and onset of physical changes. As such, different ways of modeling pubertal timing represent different points in the pubertal process. Common metrics of pubertal timing include regressing pubertal status (development) on age and using the residuals as an indicator of timing [5]. Another common metric in females is age of menarche, or age of first menstruation, which signals reproductive maturity [5]. This milestone reflects gonadal development and takes place relatively late in the course of pubertal development, an average of 1.5–3 years following the beginning of breast development [5].

Pubertal timing predicts a number of psychosocial and health outcomes [12–14]. In particular, earlier timing in females is related, on average, to negative developmental sequelae, including internalizing and externalizing symptoms, low academic achievement, earlier onset of sexual activity [13], and higher rates of obesity, hypertension, and Type 2 diabetes by adulthood [15]. Findings regarding early maturation have been less consistent in males [12, 16]. A mismatch between socially imposed responsibilities and cognitive capacities, alienation related to physical differences from one’s peers, and potential victimization of early-maturing girls (as opposed to boys) may be salient (for a review, see [14]).

Importantly, only limited research has explored how youth with neurodevelopmental disorders navigate puberty. Attention-deficit hyperactivity disorder (ADHD) is a neurodevelopmental disorder characterized by impairing levels of inattention and/or hyperactivity/impulsivity that begin prior to age 12 and are present in multiple settings [17]. In the USA, approximately 9.4% of children receive an ADHD diagnosis [18]. Although characterized by heterogeneity within and across individuals and over one’s lifetime, the disorder frequently persists into adulthood [19]. Recent work has highlighted that ADHD can and does occur in females, predicting a range of negative adolescent and adult outcomes across emotional, social, academic, and occupational domains (for a review, see [20]).

To understand ADHD from a developmental/lifespan perspective, it is necessary to understand when and how youth with the disorder navigate developmental transitions. Yet, research examining puberty in youth with ADHD is quite limited. Greenfield and colleagues (2014) found no significant differences in the timing of pubertal development as a function of ADHD diagnostic status or history of stimulant medication use [10]. Although a critical first step in a burgeoning literature, this investigation did not examine sex differences, had only a relatively small subsample of females with ADHD (*n* = 69), and did not explore age of menarche as an additional indicator of pubertal timing. Given that key developmental transitions (adrenarche, gonadarche) normatively occur 1–2 years earlier in females compared to their male counterparts, along with sexually dimorphic puberty-related changes in brain structure and function [4, 21], it is important to explore potential sex differences in the relation among diagnostic status, stimulant usage, and pubertal timing. Note that in this article, we discuss differences between males and females (i.e., sex differences between those who were assigned female at birth vs. those assigned male at birth), rather than gender differences (which is based on self-identification).

Investigating pubertal timing in girls with ADHD may be especially relevant given recent work on youth with other neurodevelopmental conditions. In particular, Corbett et al. found that females with autism spectrum disorder (ASD) showed earlier onset of breast [22, 23] and pubic hair [23] development than did neurotypical peers. Females with ASD also experienced an earlier age at menarche than comparison females [22]. On the other hand, there were no significant differences in the onset of genital or pubic hair development between males with and without ASD [22, 23].

An important consideration is that stimulant medication is a common treatment for ADHD, prescribed to over 60% of youth diagnosed with ADHD in the USA [18]. Stimulants increase the availability of synaptic dopamine; this process has the potential to decrease growth hormone secretion [24], with potential impacts on pubertal and growth processes. Research on the relation between stimulant use and growth (pubertal development, height, and weight) has often been male-dominated, yielding mixed results. A recent meta-analysis found a small but statistically significant negative impact of stimulant medications on adolescent height and weight—an effect that may have low clinical significance, as such differences often remit by adulthood [25]. Although one study found that males with an extended history of stimulant medication had lower height, weight, and pubertal stage compared to those without ADHD [26], other research has not identified a relation between stimulant medication use and reduced adult height or weight [27, 28] or pubertal status/timing [10] in either males or females. Therefore, we attempt to extend the work of Greenfield et al. [10] in a female sample to further investigate the unanswered question as to the impact of stimulants on pubertal growth in females.

Given the importance of further understanding pubertal processes of girls with ADHD, our goals are twofold. First, we compare the timing of puberty in adolescent females with and without ADHD, measured via both Tanner staging and age at menarche. Second, we address the question of whether a history of stimulant medication usage predicts the timing of physical development among girls with ADHD.

## Methods

### Participants

The Berkeley Girls with ADHD Longitudinal Study (BGALS) is a prospective investigation following girls with ADHD and age-matched neurotypical peers from childhood into adulthood. Between 1997 and 1999, girls aged 6–12 in the San Francisco/Bay Area were recruited to participate in the study through schools, mental health centers, pediatric practices, and direct advertisements. All girls in the ADHD group met full diagnostic criteria for inattentive or combined-type ADHD via structured interviews with parents using the Diagnostic Interview Schedule for Children-4th edition (DISC-4). Neurotypical comparisons did not meet diagnostic criteria for ADHD. For both groups, exclusion criteria were IQ < 70 and presence of a pervasive developmental disorder, psychosis, or neurological disorder. Following extensive diagnostic assessments, 140 girls with ADHD and 88 comparison girls were enrolled, group-matched for age and ethnicity (for detail on recruitment and diagnostic procedures, see [29]). The mean age at Wave 1 was 9.5. The sample was racially (53% White, 27% African American, and 9% Asian American), ethnically (11% Latina), and socioeconomically diverse. Participants were invited for prospective follow-up assessments 5 (Wave 2), 10 (Wave 3), and 16 (Wave 4) years after the Wave 1 assessment. The present analyses use data from Waves 1 and 2 only. Additional information about subsequent waves of data collection are described in [30].

Wave 2 (W2) occurred during adolescence and involved clinic-based assessments and data collected from multiple domains, sources, and informants, including information on psychosocial, cognitive, and academic functioning. Self-report data on pubertal development were also collected at W2, but not at earlier or later waves.

The retention rate between W1 and W2 was > 90% (209/228). Those failing to complete W2 did not differ significantly from retained participants on most measures, but were more likely to live in a single-parent household and to have higher levels of W1 internalizing behaviors per teacher report (they did not differ significantly on 29 other considered variables). On average, W2 data were collected 4.5 years after W1 (*SD* = 0.3); participants ranged in age from 11.3 to 18.2 years (*M*_age_ = 14.2 years). See Table 1 for demographic information.

**Table 1:** Demographic characteristics by ADHD diagnostic status

	Comparison *n* = 82	ADHD *n* = 127	Total *n* = 209
Age in years (SD)	13.97 (1.6)	14.36 (1.7)	14.21 (1.7)
Race/ethnicity *n* (%)			
Caucasian	38 (46.34%)	73 (57.48%)	111 (53.11%)
African American	23 (28.05%)	33 (25.98%)	56 (26.79%)
Latina	9 (10.98%)	15 (11.81%)	24 (11.48%)
Asian American	12 (14.63%)	5 (3.94%)	17 (8.13%)
Native American	0 (0%)	1 (0.79%)	1 (0.48%)
Income (W1) *n* (%)			
< $20 K	5 (6.10%)	10 (7.87%)	15 (7.18%)
$20-40 K	11 (13.42%)	22 (17.33%)	33 (15.79%)
$40-60 K	16 (19.51%)	26 (20.47%)	42 (20.10%)
$60-75 K	14 (17.07%)	22 (17.33%)	36 (17.22%)
> $75	33 (40.24%)	42 (33.07%)	75 (35.89%)
Missing	3 (3.66%)	5 (3.94%)	8 (3.83%)
Body mass index (SD)	20.92 (3.94)	23.05 (6.37)	22.22 (5.63)

## Measures

### Pubertal development/timing

**Tanner Stages***.* At W2 (ages 11–18), each girl was asked to self-report which of five increasingly developed line drawings most resembled her current level of physical development. Pictures range from prepubertal (1) to fully developed (5), with separate drawings for breast growth (BG) and pubic hair (PH) [8, 9]. Prior work has established the validity of self-reported Tanner staging in this age range, given moderately strong agreement with clinician-rated physical development [11, 31], including for youth with ADHD [10]. BG ratings were available for 201 participants (*n* = 121 with ADHD;* n* = 80 without) and PH ratings were available for 199 participants (*n* = 120 with ADHD; *n* = 79 without).

**Age at menarche***.* At W2, both girls and their primary caregivers (overwhelmingly mothers) were asked about whether the participant had begun menstruating and, if so, the age at which this milestone occurred. When available, self-reported age at menarche was used. If only parent-reported age of menarche was available, this value was used. Past work has established a high correlation between self- and parent-reported age of menarche [32]. Herein, the correlation between self- and parent-reported age at menarche was quite strong, *r*(146) = 0.75, *p* < 0.001, 95% CI [0.67, 0.81]. There remained a high correlation when separated by ADHD status (*ADHD*: *r*(92) = 0.74, *p* < 0.001, 95% CI [0.64, 0.82]; *Comparison*: *r*(52) = 0.77, *p* < 0.001, 95% CI [0.63, 0.86]). Overall, 161 participants had menstruated and had a valid age at menarche from at least one reporter.

### Stimulant usage

At W1 and W2, families were asked about the types and dates of medications the participant had received, including stimulants for ADHD. Girls were classified as positive for stimulant use if they either (1) reported taking stimulant medication at (or before) W1 *or* (2) reported at least 3 months of stimulant usage between W1 and W2, if and only if these dates preceded age at menarche. If menarche preceded W1 data collection by more than 6 months, girls were classified as positive for stimulant use only if they reported using stimulants for 3+ months at dates that occurred prior to menarche. If no reported age at menarche was available, any stimulant usage by W1 or any 3+ months of stimulant use between waves warranted classification as positive for stimulant use. Overall, 48 (37.8%) of girls with ADHD had no history of premenarcheal stimulant use and 79 (62.2%) had a history of premenarcheal stimulant use.

### Data analytic method

All analyses were conducted in R. Three methods were used for comparing pubertal timing between diagnostic groups (ADHD vs. comparison) and stimulant-medicated vs. non-medicated subgroups of the ADHD sample. (1) Replicating the procedures of Greenfield et al. [10], *χ*^2^ tests were used to compare the distribution of responses on Tanner Line Drawings (separately for BG and PH). (2) A common metric of pubertal timing is regressing pubertal status on age and using the residual as an indicator of pubertal timing (e.g., [5]). Larger residuals (positive) indicate earlier timing (more advanced development than same-aged peers), whereas smaller residuals (negative) indicate later timing (less advanced development than same-aged peers). Across our sample, BG and PH were separately regressed on age, and residuals were compared using *t* tests. (3) We computed *t* tests to compare age at menarche.

A power analysis was conducted using the pwr package in R [33], which confirmed that our sample size was sufficient to detect a small to medium effect for both *χ*^2^ and *t* tests with 0.8 power using a two-tailed alpha level of 0.05. Given the smaller sample size available for stimulant vs. non-stimulant comparisons within the ADHD group, medium effect sizes were detectable. Effect sizes were calculated for statistically significant differences using the psych package in R. For *χ*^2^ tests, an effect size (Cramer’s V) of 0.1 was considered small, 0.3 medium, and 0.5 large; for *t* tests, an effect size (Cohen’s d) of 0.2 was considered small, 0.5 medium, and 0.8 large [34].

## Results

### Pubertal development in girls with vs. without ADHD

#### Tanner staging: χ^2^ tests

There were no statistically significant differences in stages of breast development, $\chi$(4) = 7.01, *p* = 0.14, or pubic hair growth, $\chi (4)$=7.7, *p* = 0.10, in girls with vs. without ADHD (see Table 2).

**Table 2:** Tanner staging in girls with and without ADHD

	Comparison		ADHD		Total	
Tanner breast growth *n* (%)	*n* = 80	Age (SD)	*n* = 121	Age (SD)	*n* = 201	Age (SD)
1	2 (2.50%)	12.4 (0.5)	6 (4.96%)	12.3 (0.8)	8 (3.98%)	12.3 (0.7)
2	4 (5.00%)	12.1 (0.3)	7 (5.79%)	12.4 (1.2)	11 (5.47%)	12.3 (1.0)
3	28 (35.00%)	13.1 (1.4)	26 (21.49%)	13.2 (1.4)	54 (26.87%)	13.2 (1.4)
4	32 (40.00%)	14.6 (1.2)	46 (38.02%)	14.8 (1.3)	78 (38.81%)	14.7 (1.3)
5	14 (17.50%)	15.4 (1.4)	36 (29.75%)	15.4 (1.6)	50 (24.88%)	15.4 (1.5)

Tanner pubic hair *n* (%)	*n* = 79	Age (SD)	*n* = 120	Age (SD)	*n* = 199	Age (SD)
1	5 (6.33%)	12.0 (0.4)	6 (5.00%)	12.1 (0.8)	11 (5.53%)	12.1 (0.6)
2	8 (10.13%)	12.5 (1.0)	6 (5.00%)	12.4 (1.0)	14 (7.04%)	12.5 (1.0)
3	10 (12.66%)	13.0 (0.8)	23 (19.17%)	13.4 (1.4)	33 (16.58%)	13.3 (1.2)
4	34 (43.04%)	14.1 (1.5)	36 (30.00%)	14.2(1.3)	70 (35.18%)	14.2 (1.4)
5	22 (27.85%)	15.3 (1.3)	49 (40.83%)	15.4 (1.6)	71 (35.68%)	15.4 (1.5)

Later Tanner stages indicate more advanced physical development. $\upchi$ ^2^ tests did not identify significant differences in the distribution of Tanner stages for breast growth (BG) or pubic hair (PH) between girls with/without ADHD

### Residuals

Residuals did not differ significantly between girls with and without ADHD for either breast growth, *t*(188.55) = − 0.44, *p* = 0.66, or pubic hair growth, *t*(166.72) = − 0.58, *p* = 0.56.

### Age at menarche

Approximately, 70% of girls without ADHD and 82% of girls with ADHD had begun menstruating by W2. The average age of the subset of participants who had not yet menstruated was nearly identical across groups, *t*(45.46) = 0.30, *p* = 0.77 (*ADHD*: *M*_*age*_ = 12.38, *SD* = 0.8; *Comparison*: *M*_*age*_ = 12.44, *SD* = 0.8)*.*

**Fig. 1:**
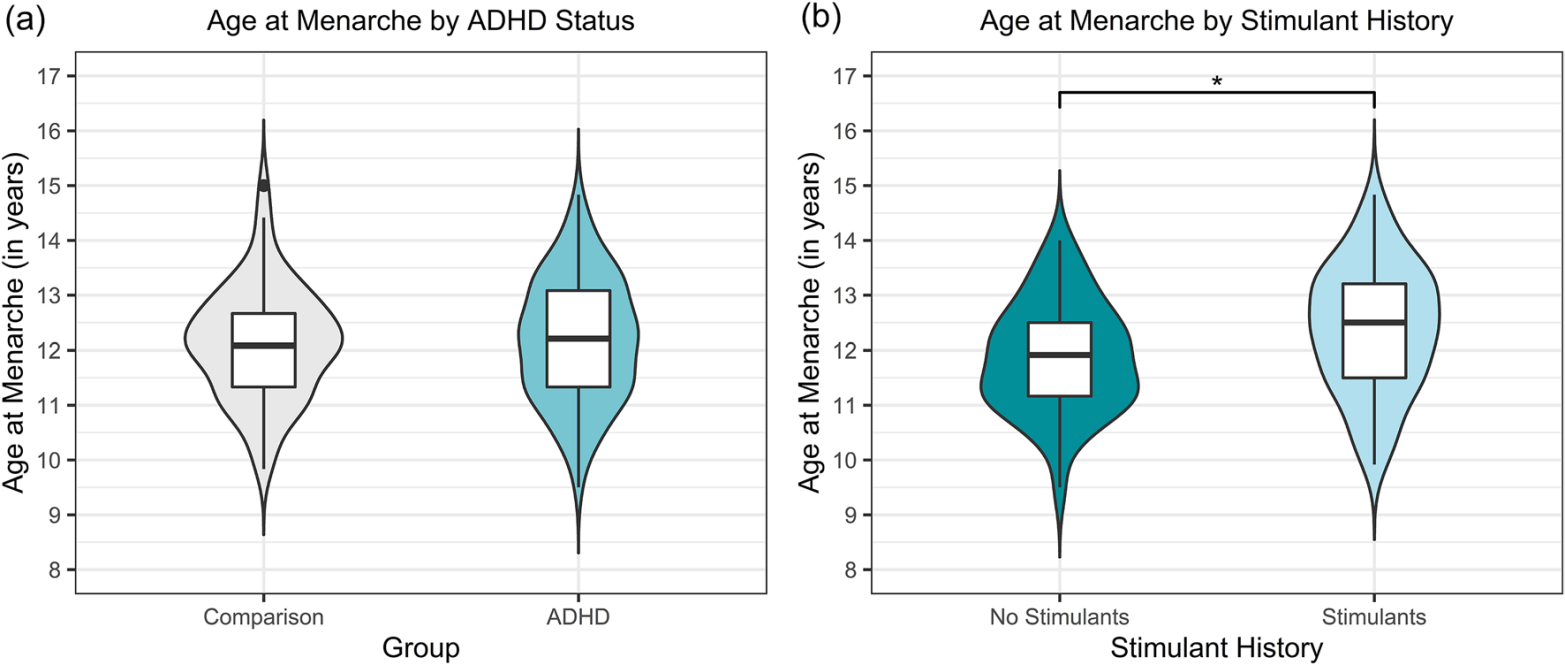
*Violin and box plots of age of menarche by group* **a** Age of menarche in girls with (*n* = 104) and without ADHD (*n* = 57) who experienced this developmental milestone; there were no statistically significant differences between groups; **b** Age at menarche among girls with ADHD who did and did not have a history of stimulant use; girls with ADHD who used stimulants (*n* = 63) menstruated significantly later than those who did not (*n* = 41)

Girls with and without ADHD experienced menarche at similar ages, *t*(12.34) = − 0.33,* p* = 0.74. The average age of menarche for girls with ADHD was 12.18 years (*SD* = 1.12) vs. 12.13 years for the neurotypical comparison sample (*SD* = 1.03) (see Table 3 and Fig. 1).

**Table 3:** Menarche in girls with and without ADHD

	Comparison *n* = 82	ADHD *n* = 127	Total *n* = 209
Ever menstruated? *n* (%)			
No	25 (30.49%)	23 (18.11%)	48 (22.97%)
Yes	57 (69.51%)	104 (81.89%)	161 (77.03%)
Avg. age at menarche (SD)	12.13 (1.03)	12.18 (1.12)	12.16 (1.09)
Avg. age of those who have not yet menstruated (SD)	12.44 (0.8)	12.38 (0.8)	12.41 (0.8)

### Pubertal development in girls with ADHD with vs. without a history of stimulant use

#### Tanner staging: *χ*^2^ tests

There were no statistically significant differences in breast growth, $\chi$(4) = 3.18, *p* = 0.53, or pubic hair development, $\chi$(4) = 1.47, *p* = 0.83, between girls with ADHD who did vs. did not have a history of stimulant usage (see Table 4).

**Table 4:** Tanner staging in girls with ADHD with and without a history of stimulant use

	No stimulant use		Stimulant use	
Tanner breast growth *n* (%)	*n* = 45	Age (SD)	*n* = 76	Age (SD)
1	1 (2.22%)	13.58 (NA)	5 (6.58%)	12.07 (0.48)
2	3 (6.67%)	12.22 (1.47)	4 (5.26%)	12.56 (1.17)
3	11 (24.44%)	13.08 (1.06)	15 (19.74%)	13.36 (1.67)
4	14 (31.11%)	15.29 (1.45)	32 (42.11%)	14.52 (1.23)
5	16 (35.56%)	15.71 (1.67)	20 (26.32%)	15.08 (1.5)

Tanner pubic hair *n* (%)	*n* = 44	Age (SD)	*n* = 76	Age (SD)
1	3 (6.82%)	12.31 (1.16)	3 (3.95%)	11.83 (0.08)
2	2 (4.55%)	12.25 (1.18)	4 (5.26%)	12.54 (1.11)
3	8 (18.18%)	13.89 (1.44)	15 (19.74%)	13.13 (1.32)
4	11 (25.00%)	14.08 (1.32)	25 (32.89%)	14.23 (1.26)
5	20 (45.45%)	15.82 (1.68)	29 (38.16%)	15.14 (1.46)

Later Tanner stages indicate more advanced physical development. $\upchi$ ^2^ tests did not identify significant differences in the distribution of Tanner stages for BG or PH between girls with ADHD who did and did not have a history of stimulant use

### Residuals

There was no significant difference in pubertal timing (residuals) between girls with and without stimulant use in terms of breast growth, *t*(100.26) = − 0.13, *p* = 0.89, or pubic hair, *t*(82.60) = − 0.97, *p* = 0.33.

### Age at menarche

Girls with ADHD who had not taken stimulants had an average age of menarche of 11.87 years (*SD* = 0.99), compared to 12.39 years for those with a history of stimulant use (*SD* = 1.16). This difference was statistically significant, *t*(94.70) = − 2.45, *p* = 0.016, 95% CI [− 0.94, − 0.10], and represents a small (nearly medium) effect size, *Cohen’s d* = 0.48, 95% CI [0.06, 0.89] (see Table 5 and Fig. 1).

**Table 5:** Menarche in girls with ADHD with and without a history of stimulant use

	No stimulant use *n* = 48	Stimulant use *n* = 79
Ever menstruated? *n* (%)		
No	7 (14.58%)	16 (20.25%)
Yes	41 (85.42%)	63 (79.75%)
Avg. age at menarche (SD)	11.87 (0.99)	12.39 (1.16)
Avg. age of those who have not yet menstruated (SD)	12.58 (0.98)	12.29 (0.7)

In post hoc analysis, we explored whether participant BMI differed as a function of stimulant history, given that higher BMI correlates with earlier age at menarche [32], and a common side effect of stimulants is appetite suppression [25]. Girls with ADHD who had taken stimulants showed significantly lower W2 BMI z-scores (for age and sex, based on WHO growth references [35]) than those without a history of stimulant use, *t*(91.39) = 2.37, *p* = 0.02, 95% CI = [0.10, 1.12], *Cohen’s d* = 0.44 (*No stimulants: M*_*BMI*_ = 24.88, *M *_*z-score*_ = 1.14; *Stimulants: M*_*BMI*_ = 21.95, *M *_*z-score*_ = 0.53). There were no significant differences in height as a function of stimulant use *t*(91.16) = -1.33, *p* = 0.19 (*No stimulants: M *_*height*_ = 5.21ft; *Stimulants: M *_*height*_ = 5.26ft)*.*

## Discussion

Adolescence is a critical period of growth, change, and maturation. Many of the physical and psychological changes associated with this period are a consequence of key pubertal transitions, which typically occur 1–2 years earlier in females than males [5, 6]. Although prior work has found no significant differences in the timing of puberty in youth with vs. without ADHD [10], investigations of youth with other neurodevelopmental disorders highlight the importance of exploring sex differences [22]. Our goal was therefore to examine the timing of pubertal development in females with ADHD, a population that has been historically understudied [20]. Using three methods of comparing pubertal timing, we examined (1) whether the timing of puberty differs between females with and without ADHD and (2) whether prepubertal stimulant use among females with ADHD predicts differences in pubertal timing.

Overall, we found no evidence that pubertal timing differs as a function of ADHD status across any of our three metrics. This pattern extends work by Greenfield et al., who similarly found no significant group differences (but did not explore sex differences) [10]. We also introduce two additional common metrics of pubertal timing not used by Greenfield and colleagues—residuals of pubertal status regressed on age, and age at menarche—which further support null results in our all-female sample.

These findings suggest that females with ADHD mature physically at a similar time as their peers without the disorder. Moreover, recent evidence in a non-clinical sample finds that inattention symptoms remain relatively consistent across pubertal stages [36]. We note that physical maturation of secondary sexual characteristics sends social signals, influencing responses by peers and adults and providing new opportunities, responsibilities, and challenges [7]. As such, the disparity between expectations based on physical appearance and continued difficulties with attention, self-, and emotion regulation may pose particular challenges for youth with ADHD as they navigate the social terrain of adolescence. Overall, girls with ADHD should receive the same instruction and education about their changing bodies and development as their peers without the disorder.

Within the ADHD sample, two indicators of pubertal timing related to Tanner staging did not differ significantly as a function of premenarcheal stimulant use. Such null effects replicate the work of Greenfield et al. [10] and extend it to an all-female sample using an additional measure of pubertal timing (residuals). Yet, we did find a significant relation between stimulant use vs. non-use and age at menarche, a key indicator of pubertal timing. Girls with ADHD who had no history of premenarcheal stimulant use had an earlier age of menarche than those with a history of stimulant use, with an effect size of nearly medium.

In post hoc analysis, we explored whether BMI differed between the stimulant and non-stimulant subgroups of girls with ADHD. Prior work finds that higher BMI is correlated with earlier age at menarche [32]. Stimulants often suppress appetite, which may contribute to lower BMI during/following treatment with these medications, at least over the short term [25]. W2 BMI z-scores were higher in the non-stimulant than the stimulant group, although we did not find significant differences in height. It is important to note that BMI was measured at W2, which was post-menarche for those for whom age at menarche was available. Further, achieving menarche is itself associated with weight gain and BMI increases among normal/underweight girls [32]. As such, claims about mediation or causal mechanisms cannot be made.

Our study is naturalistic, without random assignment of stimulant medication for girls with ADHD. It could thus be the case that non-receipt of stimulants prior to menarche is a consequence of early menarche rather than a cause. For instance, the decision to put one’s daughter on stimulants at age 12 would be post-menarcheal if she were an early maturer, but premenarcheal if she were a late maturer. As such, the relation between stimulant use and age at menarche could be spurious rather than causal. Other factors, such as potential racial differences in both the likelihood of pharmacological treatment for ADHD [18] and the timing of menstruation [32] could be at play.

More broadly, there is inconsistent and incomplete knowledge of the long-term impact of stimulants on physical and neurological development. Stimulant use in youth with ADHD yields reduced symptoms and impairment for a large number of individuals, along with potential side effects (for a review of treatment guidelines for ADHD, see [37]). Although there are concerns that the increased synaptic dopamine yielded by stimulants decreases growth hormone secretion [24], other studies have found no clear evidence that stimulant use alters gonadal function [38] or cerebral cortex growth [39] in adolescents with ADHD. Future work should examine whether the effectiveness of stimulant medication changes across the pubertal transition or with the hormonal shifts that occur across the menstrual cycle.

We note several limitations. First, we measured pubertal status using self-reported Tanner staging but do not have ratings by clinicians. Although self-reported Tanner staging is considered valid and shows moderate agreement with other raters [31], this is an imperfect measure and incorporates adolescents’ perceptions of their own development, introducing possibilities for error. As well, Tanner staging examines the development of secondary sex characteristics (pubic hair, breast growth), but does not capture changes in other physical features, such as acne, height (growth spurt), or body odor. As such, we cannot speak to the timing of these processes. Second, the age range of the sample when reporting pubertal development was relatively wide (11–18). Because most girls reach full maturation according to Tanner staging by age 14 or 15 [40], our methods may not have been the ideal metric of pubertal timing for older youth, as residuals of pubertal status on age may no longer reflect *when* they developed. Similarly, we have data on age at menarche only for those who have already reached this developmental milestone; thus, data were not available for the minority of participants who were either young or late developers (*n* = 48), who could differ from other participants. Third, our measure of stimulant usage was retrospective, lacking precise detail on exact dates of medication use. Moreover, we did not have baseline data on dosage or duration of stimulant use during childhood, which could be related to its impact on pubertal maturation. Further work in samples with more detailed tracking of medication history will be crucial in this regard. Finally, as the majority of girls with ADHD had a history of stimulant use prior to menarche, there was a limited subsample of girls with ADHD for whom this was not the case (*n* = 48), potentially limiting statistical power to identify a true effect.

In summary, via data from the largest and longest-running study of the lifespan development of girls with ADHD, we found that girls with ADHD reach developmental milestones, including menarche, at similar ages to their peers without the disorder. To our knowledge, this is the first prospective study to examine and compare age of menarche in girls with and without ADHD. We also found that girls with ADHD who took stimulants did not differ in the age at which they reached developmental milestones assessed via Tanner staging—but they menstruated, on average, around 6 months later than those who did not use stimulants. We postulate that this finding may be related to a lower BMI in girls with stimulant ADHD. There may be other reasons. Again, given the naturalistic nature of this investigation, causal mechanisms cannot be inferred.

Adolescence is a key period for risk and resilience, filled with many changes that are both biological and social in nature. As such, it is critical to characterize the pubertal transition, including but not limited to its timing, for girls with ADHD, to promote positive adjustment in adolescence and beyond for this understudied population.

## Data Availability

Questions about data availibility can be directed towards the corresponding author.
